# Empowering Personal Trainers to Work with Individuals with Disabilities to Improve Their Fitness

**DOI:** 10.3390/ijerph21080999

**Published:** 2024-07-30

**Authors:** Cassandra Beattie, Aspen E. Streetman, Katie M. Heinrich

**Affiliations:** Department of Kinesiology, Kansas State University, Manhattan, KS 66506, USA; aestreetman@ksu.edu (A.E.S.); kmhphd@ksu.edu (K.M.H.)

**Keywords:** fitness professionals, adaptive, education, experience, physical activity, barrier, facilitator

## Abstract

The benefits of regular physical activity for individuals with disabilities (IWDs) are well recognized. Nonetheless, IWDs report several barriers to physical activity participation, including limited access to qualified and experienced personal trainers. Limited research exists from personal trainers’ perspective. This qualitative study aimed to examine the experiences of personal trainers who successfully improve the fitness of IWDs. Individual interviews were conducted with ten personal trainers, focusing on educational and occupational background, experiences training IWDs, and experiences training IWDs within gyms. Thematic analysis produced five themes: (1) personal trainers working with IWDs need specialized education and extensive, often multidisciplinary, experience; (2) personal trainers are most successful when they have the opportunity to work with IWDs who have a diverse range of disabilities and differing expressions of each; (3) a robust network between personal trainers and allied healthcare providers is necessary to support IWDs; (4) access to physical activity is enhanced when trainers manage resources appropriately; and (5) personal trainers can empower IWDs to be advocates for their physical activity needs. Future research could examine the effects of an adaptive hands-on educational intervention among personal trainers to enhance IWDs’ health and fitness.

## 1. Introduction

One in four United States (U.S.) adults has or will have some form of disability that impacts their mobility, cognition, independent living, hearing, vision, or self-care in their lifetime [1]. Disability is any condition and impairment of the body or mind that impedes an individual from completing certain activities or interacting in normal daily activities, including physical activity [2]. Individuals with disabilities (IWDs) who regularly participate in physical activity have a reduced risk of chronic health conditions, improved physical and mental health, increased feelings of empowerment, and a greater sense of social inclusion [3,4,5]. However, despite clear evidence describing the benefits of physical activity, most IWDs are not physically active [4,5].

Gyms are locations where IWDs can prioritize their physical activity. Gym-based physical activity is considered exercise, a subset of physical activity that is planned, structured, and repetitive [6]. Despite gyms’ focus on prioritizing exercise, barriers persist in these locations. Gym-related barriers to IWDs’ exercise behaviors include built environment issues (e.g., lack of open space, inaccessible doors, lack of ramps, slick surfaces), inaccessible gym equipment, facilities not meeting the Americans with Disabilities Act guidelines, and a lack of qualified fitness professionals (e.g., personal trainers) [5,7,8,9,10]. Personal trainers play a unique role in supporting the health and wellness of IWDs by helping them to engage in exercise behaviors [5,11]. Personal trainers accomplish this by helping IWDs set clear, individualized, meaningful goals and motivating their clients to achieve them [11]. IWDs’ training goals often involve developing more physical independence [11]. For example, many IWDs may have the goal of walking again, independently transferring in and out of their wheelchair, eventually attending the gym alone, or feeling more confident at the gym and other locations [5,11]. Trusting relationships develop between personal trainers and their clients, who provide safety nets for IWDs to vocalize what needs are still not being met within their facility [5,11]. The relationships personal trainers create with their clients also provide peer support, improve the social inclusion of IWDs in the gym, and aid their comfort in exercising within these spaces [5,11].

Despite personal trainers’ positive role in the health and wellness of IWDs, many IWDs report negative experiences with their trainers [5,7,9,12]. These negative experiences may stem from a lack of knowledge, skill, and confidence to work with IWDs. Training IWDs requires knowledge and experience in “adapting” exercise (i.e., modifying exercise selection and programming based on limitations or impairments stemming from their disability [6,13]. Limited certifications include education on adaptive skills and hands-on experience to produce qualified and confident trainers to work with IWDs [5,9,12,14]. The limited availability of specialized certifications leads to few well-equipped trainers who can be employed, contributing to the negative experiences and barriers reported by IWDs at gyms [5,14].

Although a growing body of literature has explored the barriers to exercise participation from the viewpoint of IWDs, limited research is available from the perspective of personal trainers. Three recent qualitative studies explored the beliefs and experiences of personal trainers working with IWDs [5,9,11]. The authors found that personal trainers struggled with managing the pre-existing attitudes of other gym users, limited knowledge of exercise opportunities for IWDs, cost restraints, built environment constraints, limited transportation resources, lack of accessible equipment, lack of disability-specific training for gym/fitness staff, and a lack of collaboration with healthcare providers [5,8,9,11,15,16]. The most impactful barriers were a lack of disability-specific training for personal trainers, cost restraints, and a lack of collaboration with healthcare providers [5,8,9,16]. Personal trainers’ barriers impacted their ability to train IWDs effectively and reach IWDs needing their services [5,8,9,16].

Although these studies provide valuable observations into the barriers personal trainers face when enhancing IWDs’ health using exercise, further research is needed from the perspectives of personal trainers who have overcome barriers to training IWDs to improve the health and fitness of IWDs. Therefore, this study aimed to examine the experiences of personal trainers who successfully worked with IWDs.

## 2. Materials and Methods

To improve the health and fitness of IWDs, an online qualitative study was conducted to explore personal trainers’ experiences working with IWDs. Study participants were recruited through nonprofit organizations specializing in adaptive exercise for IWDs by sharing study flyers displaying a QR code and Bitlink to the screening survey for eligibility. Of the twelve personal trainers who clicked the screening survey, eleven were eligible to participate, and ten did so. To be eligible to participate, participants had to be at least 18 years of age or older, a US resident, English-speaking, have an active personal trainer certification or been actively certified within the past three years, and if self-identifying as having a disability, having their disability diagnosis for at least one year. The inclusion criteria dictated that participants were certified personal trainers or had been certified in the last three years. Trainers also needed to have trained IWDs. These criteria were created to ensure we gathered data about the certification they completed and their training experiences, which were recent enough to minimize recall errors. Participants who identified as having a disability were required to have had this diagnosis for at least one year to ensure that they were able to accumulate lived experiences. Both criteria combined worked to enhance the representativeness of the experiences of personal trainers who work with IWDs, including those with disabilities themselves. Semi-structured individual interviews were conducted online via Zoom. The interviews generally lasted 45 min. Study participation was voluntary, and USD 50 was provided as compensation for their time. The study received IRB approval from the university’s Institutional Review Board (approval #11364, on 24 October 2022).

Upon completing the screening survey, eligible individuals were contacted by a research team member to schedule an interview at an agreeable time for the participant. Before the interview began, each participant electronically signed an informed consent form acknowledging that the interview would be recorded but that no identifying information would be retained. The Model of Functioning and Disability loosely informed the 14 base interview questions (see Appendix A) [17]. This model describes the complex interaction between environmental and personal factors resulting in behavior patterns [17]. Expert review was then utilized to ensure the interview questions aligned with the study’s purpose. Five disability experts (i.e., physical therapists, organization leaders, and coaches), many of whom had also had a disability and all of whom regularly worked with IWDs, provided valuable insights to refine the interview questions. After their feedback was incorporated, they were asked for additional input on the final questions. Participants were asked to describe their background as a personal trainer (e.g., degrees, certifications, years of experience, etc.), experience working with IWDs, knowledge of adaptive training, adaptive friendliness of their gym, and knowledge of training programs for trainers or clients. The primary researcher regularly met with research team members throughout the interviews to assess the data saturation (i.e., when no new emerging data/themes appeared). After completing seven interviews, the research team felt they were nearing data saturation (i.e., the point in qualitative research when gathering more data no longer yields new insights or information relative to the research question [18]). More specifically, after seven interviews, the researchers were able to consistently identify recurring ideas from each trainer, such as the need for specialized education and the accumulation of hands-on-experience with IWDs. Three more interviews were completed to ensure data saturation. Upon completing ten interviews, the research team agreed that sufficient data had been gathered to develop a comprehensive understanding of the phenomena and that data saturation had been achieved [18]. Additionally, previous research describes 10–12 interviews as an acceptable benchmark for qualitative interview research [19]. Each interview was transcribed verbatim. Member checking ensured accuracy, and the recorded responses correctly reflected the participants’ experiences.

Upon completing the individual interviews, participants received a link via email to a follow-up questionnaire using Qualtrics (Provo, UT, USA). The 25-question follow-up questionnaire gathered demographic data, gym-use information, and a final opportunity for additional feedback while minimizing the participant burden by allowing participants to provide this information when convenient. If identifying as having a disability, participants were asked to specify what kind of disability they had (i.e., congenital and/or acquired) and the specific category/type of the disability (i.e., traumatic brain injury, spinal cord injury, other [specified], etc.). Participants were asked to specify the type of gym they attend or attended (i.e., CrossFit gym, adaptive gym, traditional gym, or other). Participants were also asked to specify that they were actively working as a personal trainer. Two rating questions asked participants to rate their overall satisfaction with their gym and its trainers. An additional two rating questions asked participants to rate the adaptive friendliness of their gym. A ten-point Likert scale was utilized where zero was least satisfied or not adaptive friendly, and ten was completely satisfied or very adaptive friendly. Lastly, participants were asked an open-ended probing question asking for additional experiences, barriers, facilitators, or suggestions they had to share post-interview. All ten individual interview participants completed the follow-up questionnaire (100% response rate). Notably, the follow-up questionnaire was not a quantitative arm in a mixed-methods study; it allowed the research team to gather demographic data for this qualitative study.

Trustworthiness was fulfilled through credibility and dependability by applying researcher triangulation and an imperative, definitive research process to examine and produce themes (i.e., a comparative method) [20]. Researcher triangulation was achieved by using two coders, a primary and a secondary coder [20]. Both coders employed Taguette, an open-source qualitative data analysis software, to facilitate the coding [21]. The secondary coders familiarized themselves with all the interview transcripts and coded some (approximately 33%) of the interviews. A comparative method was used to develop themes, consisting of an inductive, iterative thematic analysis [22,23,24]. The researchers independently coded the transcripts by highlighting words and phrases using Taguette’s highlighting feature that addressed the study’s research question. Both coders iteratively refined the codes until consensus was achieved. The primary and secondary coders regularly compared codes and confirmed that similar ideas and concepts were being interpreted. The researchers also returned periodically to previously analyzed data and applied any relevant new codes as they emerged. After all the transcripts had been coded, both coders met to ensure the individual code meaning was clear and similar codes were combined, yielding 105 unique codes. All 105 codes were used to form 12 categories based on commonalities. Next, the researchers returned to the literature to assist in theme development [22]. Five themes representing the 12 categories were developed. These themes were consistent with and added to previous research. This process was iterative, repeatedly moving between the participants’ responses, codes, and categories to capture the participants’ experiences accurately. A negative case analysis was completed with no conflicting viewpoints across the five identified themes [25].

Descriptive statistics were calculated using SPSS version 27 (IBM, Armonk, NY, USA). The mean ± standard deviation (M ± SD) and frequency were calculated for all the data.

## 3. Results

The interview participants (*n* = 10) were aged 18 to 54, and 60% were between 35 and 44. The majority were female (60%). Only 10% of participants had an acquired disability, while 90% reported no disability. Most participants (80%) reported IWDs as their primary training population, while 20% reported only training some IWDs. Participants reported primarily training IWDs in adaptive gyms (40%), followed by traditional gyms (30%), CrossFit gyms (20%), and a combination of gyms/home gyms (10%). On average, participants ranked their gym as adaptive-friendly (7.8 ± 2.3). Although all the participants were certified personal trainers, some worked in other complementary occupations, as shown below in Table 1. Table 1 also displays the educational background of all the participants.

### 3.1. Individual Interview Findings

Thematic analysis of the interviews resulted in five prominent themes: (1) personal trainers working with IWDs need specialized education and extensive, often multidisciplinary, experience; (2) personal trainers are most successful when they have the opportunity to work with IWDs who have a diverse range of disabilities and differing expressions of each; (3) a robust network between personal trainers and allied healthcare providers is necessary to support IWDs; (4) access to physical activity is enhanced when trainers manage resources appropriately; and (5) personal trainers can empower IWDs to be advocates for their physical activity needs. Participant excerpts are quoted verbatim; however, brackets are used for additional clarification.

#### 3.1.1. Personal Trainers Working with IWDs Need Specialized Education and Extensive, Often Multidisciplinary, Experience

Personal trainers working with IWDs require specialized education and extensive multidisciplinary experience. While most personal trainers made positive remarks regarding their comfort in training IWDs, they emphasized the need for specialized training and hands-on experience. “It doesn’t matter what your personal training background is [e.g., general certification], but you want to get the specialty training for the adaptive population”. A general personal training certification is the first step toward becoming qualified to work with IWDs. Still, specialized certifications provide additional education on modifying exercises and programming. One participant who assists in training trainers described their approach, “We teach trainers how to work with all kinds of limitations and…we talk about intellectual disability and neurological [disability] and shift our focus into more about cueing”.

However, education alone is insufficient. Most participants noted the need for hands-on experience with IWDs. Trial and error is often the only way to determine the best training fit for their clients’ limitations, capabilities, and needs. One trainer explained, “I would say a lot of my education with the adaptive community has been from in-person trial and error… I would say that’s where I get most of my knowledge… Being hands-on is the best way to learn”. Without hands-on experience, many trainers felt nervous or uncomfortable training IWDs despite having additional education. One trainer said,

You can take a class, but you will still be nervous and uncomfortable until you get the hands-on experience… Nothing really prepares us; each injury, like a spinal cord injury, is all different. My education helped a lot, but not everyone has that.

Working with IWDs required a case-by-case approach. Many individuals may have the same disability yet different symptoms, emphasizing the need for trainers to not only gain extensive, often multidisciplinary, experience working with IWDs but also aim to gain experience with a diverse population of IWDs.

#### 3.1.2. Personal Trainers Are Most Successful When They Have the Opportunity to Work with IWDs Who Have a Diverse Range of Disabilities and Differing Expressions of Each

Participants reported extensive experience working with a wide range of disabilities, including neuromuscular, neurological, physical, and intellectual conditions, both acquired and congenital. They emphasized three levels of diversity in disabilities:

1. Differences between disability types,
2. Variations among individuals with the same disability,
3. Day-to-day fluctuations within a single individual’s condition.

One trainer highlighted the diversity of spinal cord injuries, saying, “They are all so very different even at the same level of injury: the way they present, the way they’re able to move, you know which muscles are working, everyone is so different, and that’s just one disability”. Another trainer stressed the uniqueness of each case, particularly with neurological disabilities, “If you’ve met one person with a brain injury, you’ve met one person with a brain injury… There’s such a diverse population and such a diverse range of limitations”.

This diversity often leads to initial nervousness among trainers when working with a new client, even with extensive experience.

I always find myself nervous to work with the person [IWD] in an exercise setting…I don’t feel confident until I’ve learned the person… because you can have two people with spinal cord injuries, and their capabilities are vastly different from one another.

The trainers described the necessity of an individualized approach, taking time to know their client’s specific limitations and capabilities. “Every person is kind of an N of one,” where everyone is treated as having new and unique limitations that the trainer must take time to explore. Getting to know each individual and their specific limitations and capabilities allowed the trainers to create challenging training programs that met the needs of IWDs while providing a platform to create fun, healthy relationships around physical activity. One way the trainers gained access to a more diverse population of IWDs was by actively participating in referral networks with allied healthcare providers.

#### 3.1.3. A Robust Network between Personal Trainers and Allied Healthcare Providers Is Necessary to Support IWDs

Collaboration between personal trainers and healthcare providers benefits IWDs. However, many trainers discussed barriers to collaboration. They noted the burden of fortifying the trainer–healthcare provider relationship frequently falls on the trainer, though “some physicians, health groups, and primary care groups…do a good job of connecting their patients [IWDs] with [preventive measures for their disease management]”. The trainers acknowledged that it can be difficult for healthcare providers to find qualified personal trainers to refer their patients to. One participant suggested “reaching out to your local physical therapy departments and saying, ‘Hey, this is what I provide. This is what I’m looking to do”. Other participants noted the value of hosting meet-and-greet events where healthcare professionals and IWDs can meet trainers, thus creating a robust support network for IWDs. All the trainers agreed that referral networks must be fortified to promote physical activity and improve IWDs’ fitness. Without these networks, “[IWDs] are just left to freely find them [personal trainers] and be on their own”.

#### 3.1.4. Access to Physical Activity Is Enhanced When Trainers Manage Resources Appropriately

Participants identified cost as a significant barrier to IWDs accessing fitness opportunities, including expenses for training, gym memberships, and specialized equipment. Despite financial constraints, trainers have developed strategies to make training more accessible for IWDs:

1. Grant Funding: Many nonprofit organizations provide grants to support one-on-one training, group classes, and equipment costs. As one trainer noted, “Find a grant to pay for a class, [and] to pay the coaches... I think if you can make it free to start, it helps or really lowers the cost”.
2. Donation-Based Training: Some trainers offer free, donation-based classes. One trainer shared, “We now have classes for these athletes... completely donation-based, completely free for these athletes. It’s a great way for them to feel included in a gym”.
3. Gym-Initiated Solutions: Trainers advocate for gyms to implement the following:
  - Purchase of specialized equipment (tax-deductible for the gym)
  - Sliding scale membership systems
  - Scholarship opportunities
  - Member sponsorship programs

As one trainer explained, “Buying your athlete’s equipment for the gym comes off your taxes... We have a founding member program that if you, as a member of the gym, want to pay for somebody else’s [membership] in the gym, you can”.

#### 3.1.5. Personal Trainers Can Empower IWDs to Be Advocates for Their Physical Activity Needs

Personal trainers play a critical role beyond exercise training, teaching IWDs to advocate for themselves by inspiring confidence, creating safe spaces and trusting relationships, and stepping into the role of advocate when required. Personal trainers should aim to make IWDs confident in their ability to train alone. One trainer shared, “My goal is to make them [IWDs] as independent as possible with these exercises so they can be doing them even after our training sessions”. Another explained how creating a safe space within the gym creates opportunities, saying, “Sometimes, it takes a person being present in that space [the gym]… to advocate for themselves”. This is why trainers must make “sure that they [IWDs] know they don’t have to be afraid… It’s having that safety component in the relationship”. Another participant added that part of empowering IWDs is reminding them that they are more than their disability. “You’re going to have to advocate for yourself big time, and you’re still a whole person, and you’re not your injury [or disability]”. Nonetheless, participants suggested that trainers sometimes must advocate for their clients. For example, one trainer recounted how many IWDs felt underrepresented within the gym, so this trainer advocated for increased representation among members and staff. “Representation is huge…Are we having people with disabilities working in these [gym] spaces?... Are we seeking to employ people who have disabilities?” Safe, trusting relationships between personal trainers and IWDs provided a safe space for IWDs to have a voice. Providing IWDs with a voice, advocating for their needs, and increased representation empowers IWDs to feel they can speak up for themselves even when training independently in the gym.

## 4. Discussion

This qualitative study aimed to enhance the health and fitness of IWDs by examining the experiences and perspectives of personal trainers who successfully train IWDs. We learned that personal trainers’ readiness to train IWDs is often lacking, making it difficult to support this group’s unique health and fitness needs. Five themes emerged from our analysis. These themes included obtaining specialized education and experience, having opportunities to work with a diverse range of IWDs, leveraging the trainer’s network to include allied health professionals, using resources effectively, and the trainer’s role in empowering clients with disabilities to be physically active.

Education and experience were two influential factors that influenced the trainers’ comfort and confidence in providing high-quality service to IWDs. The study participants agreed that a general personal training certification was insufficient to prepare trainers to work with IWDs. Several studies suggest the need to develop specialized certifications to train a diversity of IWDs safely and effectively through appropriate exercise adaptation [5,9,12,14]. Obrusnikova et al. (2021) highlight the lack of disability-related certifications and call attention to revising certification standards to better prepare trainers to provide safe, individualized, effective training for IWDs [9]. Participants agreed that recruiting poorly qualified and unconfident trainers can have adverse effects on clients with disabilities.

Many study participants pursued higher education (e.g., bachelor’s, master’s, and doctoral degrees) in addition to their personal training certification. Several participants also worked in complementary occupations (e.g., physical or occupational therapists), allowing them to gain multidisciplinary knowledge. These participants asserted that their additional education and separate occupations allow them more opportunities to gain hands-on experience and practical skill application. Previous research suggests that most IWDs feel their trainers lack sufficient knowledge and skills to train them [5,7,8,9]. However, the study participants suggested that additional certifications, education, and complementary occupations may be a solution to producing qualified trainers. Almost all the participants emphasized that specialized certifications and higher education were necessary, but combining knowledge with experience was even more critical. Participants indicated that hands-on experience was vital in developing competency in applying adaptive training principles and feeling confident to do so successfully.

Previous research notes the need for extra time and proper training to acquire the knowledge, skills, and experience to provide high-quality training to IWDs [9]. Many IWDs have reported negative training experiences with their trainers due to their limited knowledge and experience [5,9]. Participants discussed the knowledge–proper application gap, which could result in adverse client and trainer experiences. Therefore, participants suggested that trainers take extra time to gain hands-on experience with IWDs to improve the trainer’s confidence and the overall training experience. Given the range of disabilities, participants also discussed the case-by-case approach to disability, fueling the need for more experience with a diverse spectrum of disabilities and their unique expressions.

Understanding the unique symptomology of a client’s disability can be an overwhelming task for someone with only limited experience. Many study participants had secondary careers as health professionals, providing extensive experience working with IWDs. As such, participants recognized that many individuals might have the same disability but differing symptoms. The study participants emphasized that of the various forms of disability, many disabilities are also congenital or acquired, with some acquired disabilities having been acquired traumatically. Obrusnikova et al. (2021) found that personal trainers need further experience practicing effective communication to understand the individual range of symptoms and gain competency in identifying invisible symptoms of disability instead of the obvious physical symptoms. The study participants collectively agreed [9]. Many participants working with clients with spinal cord injuries noted the additional experience required to identify invisible symptoms. Moreover, participants also emphasized the need for experience in practicing effective communication (e.g., listening to the client describe their symptoms instead of assuming) with their client to adapt their exercise training effectively.

Without secondary careers as healthcare providers, finding opportunities to gain additional experience with IWDs may be challenging. Previous research suggests that partnering with healthcare providers may provide one avenue for experience with various unique disability cases while simultaneously providing mentorship [11]. Participants noted that partnering with healthcare providers is an effective way to gain experience working with IWDs with visible or invisible symptoms. Future research should consider studying the effectiveness of a mentoring program between personal trainers and healthcare providers in preparing trainers to work with diverse disability expressions.

Participants agreed that collaborating with healthcare providers improved the quality of training and the amount of training available to IWDs, emphasizing the need for active referral networks. However, participants discussed how the lack of active referral networks is primarily due to healthcare providers’ limited awareness of accessible training programs for IWDs. These findings align with previous research [5,9,15]. Several studies cite the limited awareness of accessible training programs because of limited communication between healthcare providers and personal trainers, with insufficient time for providers to establish these relationships [5,9,12]. One study suggested the fitness industry’s need to promote personal trainers’ roles in supporting IWDs to engage in exercise [5]. The study participants agreed and encouraged trainers to assume responsibility for establishing referral networks with allied healthcare providers by arranging meet-and-greet events for healthcare providers, IWDs, and personal trainers at their facilities [5]. Meet-and-greet events allow personal trainers to display their qualifications and excitement about working with IWDs. Future research should examine the effectiveness of meet-and-greet events in producing referral networks between trainers and healthcare providers and how this impacts IWDs’ health and fitness.

A key barrier impacting IWDs’ exercise levels is accessing cost-friendly training. Hill et al. (2023) suggested that personal trainers may be more cost-effective than allied healthcare in relation to the individual’s health and wellness. However, trainers are largely underutilized [5]. However, studies suggest that personal trainers working with IWDs face financial barriers [5,9]. Reducing training costs to accommodate IWDs requires trainers to find additional funding to be fully compensated [5,9]. Participants managed to overcome financial constraints by exploring funding resources that were available to them. Nonprofit organizations were highlighted as crucial assets supporting IWDs’ training expenses. Numerous study participants applied for grant funding from allied organizations to supplement the costs of one-on-one training, group training, and equipment. Seeking funding from allied organizations reduces one entry barrier by lessening the financial burden on IWDs and personal trainers. Future research should aim to better understand how to connect personal trainers and allied organizations supporting IWDs.

Additional studies suggested that gym management is responsible for improving awareness of their training services for IWDs while supporting the accessibility of these services [5,9,11]. Dolbow and Figoni (2015) found that although ADA guidelines mandate equal access to public facilities, including accommodations for IWDs, most gyms are not 100% ADA compliant [10]. The study participants added to this by suggesting that ADA compliance alone is insufficient. Gym personal trainers must advocate for IWDs’ needs, including affordable memberships, personal training costs, and adaptive-friendly equipment to better support their health and fitness.

The study participants agreed that personal trainer responsibilities include advocating for clients’ needs and encouraging clients to self-advocate in the gym. Hill et al. (2023) found that personal trainers represent the ethos and culture of the gym environment and can help facilitate the acceptance and inclusion of those with disabilities [5]. Moreover, Cunningham et al. (2023) found that disability inclusion in the gym is supported by staff training and a positive environment [11]. As such, participants suggested trainers advocate for more cost-friendly training options, the increased representation of IWDs in the gym, staff training, and more accessible/adaptive friendly equipment to expand the inclusion and acceptance of their gym to IWDs. Personal trainers advocating for the acceptance and inclusion of IWDs create more positive gym experiences among IWDs [5,12]. The study participants agreed and added that supporting disability inclusion increases IWDs’ confidence to train alone in the gym, increasing their overall exercise levels. Participants also believed that preparing IWDs to train alone aids their ability to self-advocate. Encouraging IWDs to self-advocate requires personal trainers to foster a safe environment to vocalize individual needs. The study participants believe that providing a safe environment to vocalize individual needs and ultimately supporting these requests empowers IWDs in the gym.

This study had several strengths. To the best of our knowledge, this is the first study to examine the experiences of trainers working with IWDs who come from diverse educational and occupational backgrounds. Our data suggest that personal trainers’ readiness to train IWDs differs based on their educational and occupational background. A further strength of the differing educational and occupational backgrounds was that it provided the perspectives of trainers working with various disabled client populations. Future research should take care to differentiate between personal trainers who have pursued additional certifications, higher education, or have complementary occupations outside of personal training. Our qualitative design involving semi-structured interviews led to data saturation, including descriptive, compelling data that cannot always be gathered quantitatively. Given the rich data, participant responses were thoroughly explored.

Several limitations were present. The study criteria required that the personal trainers had either an active training certification or were actively certified within the past three years. However, the personal trainer criteria conflicted with the disability criteria of having their diagnoses for at least one year. An individual could meet the personal trainer requirements but not the disability requirements. However, that was not the case among anyone screened for eligibility. Additionally, including trainers who met the study criteria but were not actively training IWDs may have influenced the accuracy of their responses. The small sample size may limit the generalizability of the findings, primarily since our sample was predominantly comprised of well-educated females. However, data saturation was reached, mitigating some of these concerns for the population represented in the study. We also acknowledge that our participants may not have trained IWDs full time (i.e., 40 h per week); thus, their experiences may differ from “typical” trainers. This variability in working hours may have influenced their responses compared to other full-time trainers. However, it should be noted that typical trainers often are not qualified to work with IWDs. Next, the cross-sectional nature of the current study is limited as our results were unable to analyze behavior over time. Longitudinal research will be crucial in understanding how personal trainers from differing educational and occupational backgrounds successfully overcome the barriers to training IWDs. Social desirability may have contributed to more positive results among participants. Finally, despite using an online design, the participant responses primarily came from three U.S. states. Future research should include participants from a broader range of U.S. states to better assess subjective experiences training IWDs, including those of personal trainers and adaptive sports coaches. These data could inform personal trainers and coaches seeking information on how to prepare themselves to work with IWDs and provide a better understanding of their role in the care network of IWDs.

## 5. Conclusions

Personal trainers with specialized education and industry experience shared valuable insights about working with IWDs. Participants asserted that trainers must expand their disability education through specialized certifications or higher education combined with hands-on experience. The study participants emphasized the importance of hands-on experience, with referral networks as one avenue to gain additional experience and knowledge of IWDs. Participants also indicated that personal trainers must assume responsibility for establishing these referral networks with allied healthcare providers and identify additional allied organizations (e.g., nonprofit organizations). Allied organizations can provide resources or financial relief for personal trainers working with IWDs. Financially supporting personal trainers allows more trainers to assist IWDs in meeting their exercise needs. Personal trainers must also advocate for the needs of IWDs at their respective facilities and encourage their clients to advocate for themselves. Gyms should act as enabling exercise environments; however, barriers persist regarding the readiness of trainers to work with a diverse range of IWDs. Better preparing personal trainers to provide high-quality training for a wide range of disabilities may improve IWDs’ experience with personal trainers, increase their overall exercise levels (combined and independently), and ultimately, improve their health. This information could inform multi-level interventions among personal trainers, community allies (e.g., healthcare providers or organizations), and IWDs, leading to increased levels of exercise and enhanced health and fitness.

## Figures and Tables

**Table 1:** Participants’ occupational and educational background.

Secondary Job Classification	*N*
Physical therapist	2
Occupational therapist	1
Athletic trainer	1
Speech pathologist	1
Brain injury specialist	1
Assistant professor	1
Highest Level of Education	*N*
High school degree	3
Bachelor’s degree	2
Master’s degree	1
Doctoral degree	4

## Data Availability

Data are available from the corresponding author.

## Appendix A

The individual interview questions are provided below.

1. Tell me a little bit about yourself and your experience working at a gym and/or gyms?
  - What degrees do you have?
  - What training certifications or programs have you completed?
  - What training workshops have you completed?
  - How many years of experience do you have in the field?
  - What gyms have you worked for?
2. What do you know about those with disabilities?
3. What do you know about how many of those with disabilities we have in the U.S.?
4. What do you want to know and/or learn about IWDs?
5. Explain your level of comfort for working with IWDs?
6. Explain what makes your gym welcoming (or not welcoming) for IWDs?
7. What do you think your gym could do to better facilitate exercise for IWDs?
8. Have you previously worked with any adaptive clients or IWDs, and if so, what did this work involve?
9. Have you ever adapted a movement for someone with a disability? If yes, can you provide an example of how you did this? How effective was the adaptation?
10. How could you better facilitate exercise for IWDs?
11. Are there specific training programs that have been helpful for you for working with IWDs (i.e., training regimens for IWDs)?
12. Are there specific programs that gyms should offer for trainers to work IWDs (i.e., training programs for trainers, staff, coaches)?
13. Do you believe there are any environmental gym barriers based on how they are set up that make things difficult for IWDs?
14. Do you have any final thoughts/comments/concerns/suggestions and/or experiences you would like to share?
